# Efectos del consumo de café sobre el cortisol y la alfa-amilasa salival en adultos jóvenes

**DOI:** 10.21142/2523-2754-1202-2024-197

**Published:** 2024-06-27

**Authors:** Rolando Pablo Alejandro Juárez, Armando César Celia

**Affiliations:** 1 Grupo de Investigación y Desarrollo: Saliva como Fluido Diagnóstico. Facultad de Odontología. Universidad Nacional del Nordeste. República Argentina. ropablojuarez@odn.unne.edu.ar, accelia@odn.unne.edu.ar Universidad Nacional del Nordeste Grupo de Investigación y Desarrollo: Saliva como Fluido Diagnóstico Facultad de Odontología Universidad Nacional del Nordeste Argentina ropablojuarez@odn.unne.edu.ar accelia@odn.unne.edu.ar

**Keywords:** café, saliva, alfa-amilasa, cortisol, coffee, saliva, alpha-amylase, cortisol

## Abstract

**Objetivo:**

El propósito de este estudio fue determinar los efectos del consumo de café sobre el cortisol salival (Corts) y la alfa-amilasa salival (AAs) en adultos jóvenes.

**Materiales y métodos:**

: Sesenta estudiantes universitarios sanos, consumidores habituales de café, participaron en este estudio observacional descriptivo. Los participantes se dividieron en tres grupos: G1 bajo consumo (≤ 2 tazas de café por día, n = 20), G2 consumo moderado (2-5 tazas de café por día, n = 20) y G3 alto consumo (> 5 tazas de café al día, n = 20). La autorrecolección de saliva fue por la mañana (6:30-7:30 a. m.) y por la noche (08:00-09:00 p. m.). Se analizó Corts mediante quimioluminiscencia y actividad de AAs por método cinético. El análisis estadístico de los datos se realizó utilizando la prueba t de Student y el análisis de varianza.

**Resultados:**

La muestra estuvo compuesta por 30 mujeres y 30 hombres, con edades comprendidas entre 20 y 35 años. En todos los grupos, los valores de Corts fueron más elevados por la mañana (a. m. 0,29 ± 0,19 vs p. m. 0,09 ± 0,05 µg/dl, p < 0,0001). En contraste, los niveles de AAs fueron más altos por la noche (p. m. 160,16 ± 60,42 vs. a. m. 32,79 ± 12,98 U/ml, p < 0,0001). No se detectaron diferencias significativas, en los contenidos de Corts y AAs, entre los grupos.

**Conclusión:**

El consumo de café, en condiciones no estresantes, no alteró los niveles y patrones de Corts y AAs en adultos jóvenes.

## Abstract (en)

**Objective:**

The purpose of this study was to determine the effects of coffee consumption on salivary cortisol (sCort) and alpha amylase (sAA) in young adults.

**Materials and methods:**

Sixty healthy university students, habitual coffee consumers, participated in this descriptive observational study. Participants were divided into three groups: G1 low consumption (≤ 2 cups of coffee per day, n = 20), G2 moderate consumption (2-5 cups of coffee per day, n = 20), and G3 high consumption (>5 cups of coffee per day, n = 20). Saliva self-collection was in the morning (6:30-7:30 AM) and at night (08:00-09:00 PM). sCort was analyzed using chemiluminescence and sAA activity by kinetic method. Statistical analysis of the data was performed using Student's t-test and analysis of variance.

**Results:**

The sample consisted of 30 women and 30 men, aged between 20 and 35 years. In all groups, sCort values were higher in the morning (AM 0,29 ± 0,19 vs. PM 0,09 ± 0,05 µg/dl, p < 0.0001). In contrast, sAA levels were higher in the night (PM 160,16 ± 60,42 vs. AM 32,79 ± 12,98 U/ml, p < 0.0001). No significant differences were detected, in the contents of Corts and AAs, between the groups.

**Conclusion:**

: Coffee consumption, in non-stressful conditions, did not alter levels and patterns of sCort and sAA in young adults.

## INTRODUCCIÓN

Estresores físicos o psicológicos activan dos sistemas biológicos, el eje hipotálamo-pituitario-adrenal (HPA) y el sistema simpático adrenal medular (SAM), con una variedad de respuestas fisiológicas que muestran variaciones individuales relacionadas con la salud o enfermedades futuras ^1^. Las drogas de abuso lícitas (alcohol, nicotina y cafeína) también pueden actuar como factores estresantes ^2^^,^^3^. 

La cafeína es un potenciador cognitivo ampliamente utilizado por estudiantes, jugadores de videojuegos y trabajadores del transporte que la utilizan regularmente para aumentar su estado de alerta, vigilancia, atención y tiempo de reacción ^4^. El impacto de la cafeína depende del nivel de consumo; la ingesta moderada tiene un impacto mínimo en la salud, mientras que los altos niveles de consumo pueden provocar intoxicación y efectos secundarios graves ^5^. 

En los últimos años, los investigadores y clínicos se han interesado cada vez más en los métodos de muestreo no invasivos, como las pruebas de saliva. Se ha informado que los niveles de alfa-amilasa salival (AAs) y cortisol salival (Corts) mostraron correlación con los niveles de estrés ^6^. La AAs constituye un indicador confiable de la función del sistema SAM ^7^, dado que frente a situaciones estresantes se activa el sistema nervioso simpático (SNS) y aumenta la actividad de AAs ^8^. Por su parte, el Corts es un biomarcador crítico de la reactividad del eje HPA frente a factores estresantes y se caracteriza por su amplia variabilidad intra e interindividual ^9^.

Sin embargo, la literatura no es concluyente con respecto a los efectos del café en la actividad de AAs y Corts. La mayoría de los estudios se realizaron en relación con el estrés, con dosis elevadas de cafeína o su retiro abrupto previo a la determinación, y con muestreos en entornos clínicos y de laboratorio ^3^^,^^10^^-^^12^. Además, son desconocidos los efectos del café sobre AAs y Corts en consumidores habituales evaluados mediante pruebas salivales con recolección en entornos de atención no clínica. Por lo tanto, el presente estudio examinó los efectos del café sobre el Corts y AAs en adultos jóvenes que consumían café a diario y con recolección de las muestras de saliva en el hogar.

## MATERIALES Y MÉTODOS

Se respetaron las recomendaciones para investigaciones con seres humanos y todos los participantes firmaron un Formulario de Consentimiento Informado. El protocolo experimental fue aprobado por el Comité de Bioética de la Investigación de la Facultad de Odontología de la UNNE (número de dictamen: 121-2018).

El estudio tuvo un diseño observacional descriptivo (código de identificación de proyecto investigación: 18J001, Secretaría General de Ciencia y Técnica, Universidad Nacional del Nordeste). Se realizó en condiciones de vida libre ^13^, sin alterar las actividades habituales de los participantes y con toma de muestras de saliva en ambientes no clínicos. 

Sesenta estudiantes universitarios del área de formación profesional de la carrera de Odontología de la UNNE, consumidores habituales de café, fueron reclutados para participar en este estudio. Eran del mismo año de cursada y con actividades diarias generales comparables, seleccionados mediante un muestreo por conveniencia. Se incluyeron hombres y mujeres, dado que las diferencias de sexo en la farmacocinética de la cafeína pueden alterar su metabolismo y absorción ^14^.

Los criterios de elegibilidad fueron los siguientes: adultos jóvenes sanos, usuarios diarios de café instantáneo con cafeína, que consumían al menos una taza de 250 ml por día. Fueron excluidos los participantes con problemas de salud física o psiquiátrica, y los usuarios de medicamentos o drogas que podrían afectar la interpretación de los datos de biomarcadores salivales. Además, se excluyó a quienes informaron seguir una dieta de adelgazamiento o cualquier otra dieta especial, atletas competitivos y fumadores. 

### Preparaciones de café

Desde el día de ingreso al estudio, se enseñó a los participantes cómo preparar una taza de café instantáneo. Se preparó con dos cucharaditas rasas de café (~ 4 gramos) en 250 ml de agua caliente a punto de hervir y una cucharadita rasa de azúcar refinado (~ 4,69 gramos).

### Recopilación de datos

Mediante un cuestionario preliminar, realizado en conjunto con la historia clínica, se evaluó el consumo habitual de café con cafeína durante los años anteriores (área de formación básica de la carrera de Odontología de la UNNE) y la existencia de otras fuentes de cafeína. Con esa información, los participantes se dividieron en tres grupos: G1 usuarios de café de bajo consumo (≤ 2 tazas de café por día, n = 20), G2 usuarios de café de consumo moderado (2-5 tazas de café por día, n = 20) y G3 usuarios de café de alto consumo (> 5 tazas de café al día, n = 20). 

El IMC se calculó a partir de pesos y alturas medidos en todos los participantes, como el peso en kilogramos (kg) dividido por la altura en metros al cuadrado (m)^2^. Se utilizó la categorización del IMC establecida por la OMS ^15^: bajo peso (< 18,5), normal (18,5 a 24,9), sobrepeso (25,0 a 29,9), obesidad grado I (30 a 34,9), obesidad grado II (35 a 39,9) y obesidad grado III (40 o más). 

### Recolección de saliva

Durante los 5 días anteriores a la jornada experimental, se instruyó a los participantes para que comieran alimentos similares, con una dieta baja en grasas y durmieran al menos 7 horas. Asimismo, se aconsejó evitar las bebidas alcohólicas, la cafeína cuya fuente no fuera café, el ejercicio excesivo, las situaciones estresantes y las emociones. Los días de recolección y medición fueron entre semana, con horarios de clases normales, y se evitaron los días de exámenes o con exigencias académicas no habituales. Al menos 2 horas antes de la recolección matutina y nocturna, se pidió a los participantes que se abstuvieran de comer, cepillarse los dientes, hacer ejercicio y consumir café u otra bebida que no fuera agua. 

Los participantes recolectaron muestras de saliva entera sin estimular en casa, usando salivación (babeo) pasiva ^16^. Se instruyó a los participantes que acumulen saliva en el piso de la boca y luego lo dejaran gotear del labio inferior en un recipiente plástico con cierre hermético sin conservantes. La autorrecolección fue por la mañana (6:30-7:30 a. m., aproximadamente una hora después de despertar) y por la noche (08:00-09:00 p. m.). Se indicó que las muestras de saliva se refrigeraron hasta su transporte al laboratorio. Todos los participantes cumplieron con estos criterios. 

### Determinaciones bioquímicas

El estudio se realizó en el Laboratorio de Investigaciones Científicas de la Facultad de Odontología de la Universidad Nacional del Nordeste, entre febrero y junio de 2022. Todas las sesiones de laboratorio comenzaron a las 08:00 a. m. Antes de las determinaciones, se centrifugaron a 3000 rpm durante 10 m, para eliminar mucinas y otras partículas que pudieran interferir con el ensayo. La actividad de AAs se analizó mediante un método cinético a 405 nm, con sustrato CNPG3, siguiendo el protocolo del fabricante (Kit Amilasa 405, analizador CM 250, Wiener Lab®, Argentina), y fue expresada en U/ml. El Corts fue medido utilizando inmunoensayo por quimioluminiscencia (Cobas e801, Roche Diagnóstico) y fue expresado en µg/dl. Las muestras de cada participante se analizaron por duplicado y los valores utilizados en los análisis de datos son los promedios de pruebas duplicadas.

### Análisis de los datos

Los análisis estadísticos fueron elaborados mediante el *software* InfoStat 2018 (Universidad Nacional de Córdoba, Argentina). Se realizó un análisis exploratorio de los datos, estadístico de chi-Cuadrado, coeficiente de correlación de Pearson, pruebas de t para muestras pareadas y análisis de varianza, con un nivel de significancia α = 0,05.

## RESULTADOS

La edad de los estudiantes de la muestra osciló entre un mínimo de 20 años y un máximo de 35 años, con un promedio de 24,82 años. El género se dividió en partes iguales, con un 50% de mujeres y hombres en los tres grupos de estudio. 

El IMC de los pacientes varió entre un mínimo de 18,0 y un máximo de 38,4, con un rango de 20,4. El 50% de los pacientes presentaron valores inferiores a 25,95, el 25% se encontraba por debajo de 23,1 y el restante 25%, por encima de 27,9, por lo que el 50% central abarcó 4,8 unidades. El promedio fue de 25,84, con una desviación estándar de 3,8 y un coeficiente de variabilidad de 14,69, lo que indica una variabilidad media. El coeficiente de asimetría fue de 0,63, lo que representa una asimetría positiva, y el de curtosis de 1,08 señala que la curva tiende a ser angosta y alta.

En la Tabla 1 se presentan las estadísticas descriptivas para las variables cuantitativas bioquímicas estudiadas. Los niveles de actividad matutinos de AAs fueron más bajos que los vespertinos (T = -17,10; p < 0,0001), mientras que Corts presentó contenidos matutinos más altos que los vespertinos (T = 7,52; p < 0,0001). Los coeficientes de asimetría matutinos de AAs y Corts indican curvas casi simétricas, mientras que los vespertinos revelan una asimetría positiva. Los coeficientes de variabilidad fueron altos, con valores más elevados en el Corts.

**Tabla 1:** Estadísticas descriptivas para variables bioquímicas (matutinas y vespertinas)

Variables	Me	DS	CV	Min	Max	Ra	Md	Q1	Q3	RIQ	As	K
AAs-a. m.	32,79	12,98	39,60	6,82	60,75	53,93	29,59	22,64	44,43	21,79	0,07	-0,78
AAs-p. m.	160,16	60,42	37,72	79,60	390,55	310,95	141,71	135,22	152,63	17,41	2,43	5,64
Corts-a. m.	0,29	0,19	67,28	0,05	0,84	0,79	0,23	0,15	0,42	0,27	0,97	0,45
Corts-p. m.	0,09	0,05	62,22	0,05	0,27	0,22	0,06	0,05	0,10	0,05	1,77	2,09

Me: promedio aritmético, DE: desviación estándar, Var: variancia, CV coeficiente de variación, Mín: mínimo, Máx: máximo, Ran: rango, Md: mediana, Q1: cuartil 1, Q3: cuartil 3, RIQ: rango intercuartílico, AS: asimetría, K: curtosis.

**Figura 1:**
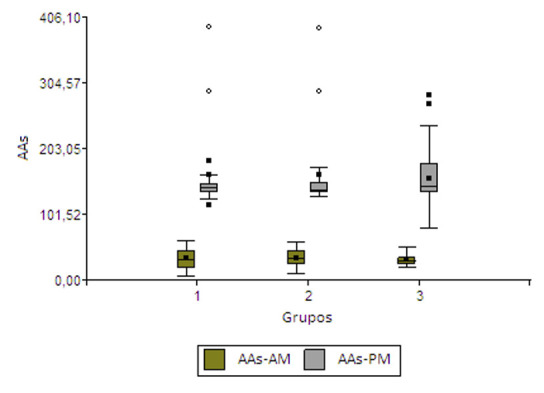
Gráficos de caja de los contenidos de AAs-a. m. y AAs-p. m. por grupo de estudio

En la Figura 1 se presentan los gráficos de caja de los contenidos de AAs matutina y vespertina por grupo de estudio. La distribución de AAs sigue el mismo patrón que la general, pues los contenidos vespertinos son más altos que los matutinos. Cuando se incluyó como única fuente de variabilidad los grupos de estudio, el análisis de la varianza y la posterior prueba de F no se pudo detectar diferencias significativas en los contenidos de AAs en ambos momentos de medición (a. m. [F= 0,04; p = 0,9600]; p. m. [F= 0,07; p = 0,9338]). Al analizar los factores grupo, género y su interacción, solamente resulta significativa la diferencia en el contenido de AAs-a. m. considerando la fuente de variación género (F= 6,86; p = 0,0114). Al incluir los factores grupo e IMC, no se detectaron efectos significativos para ninguno de los factores ni para sus interacciones. Al incluir los tres factores (grupo, género e IMC) y sus interacciones en el análisis, no se encontró efecto significativo de ninguno de los factores, solamente en la interacción grupo por género para AAs-p. m. (F= 7,95; p = 0,0003). La prueba de Duncan indicó que dicho contenido era mayor en los pacientes masculinos.

**Figura 2:**
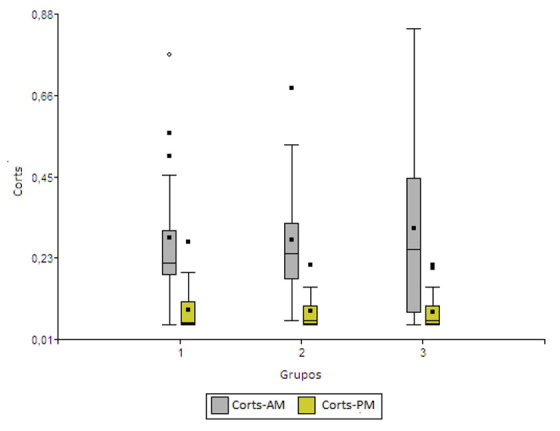
Gráficos de caja de los contenidos de Corts-a. m. y Corts-p. m. por grupo de estudio

En la Figura 2 se presentan los gráficos de caja de los contenidos de Corts matutino y vespertino por grupo de estudio. La distribución de Corts sigue el mismo patrón que la general, los contenidos matutinos son más altos que los vespertinos. Cuando se incluyó como única fuente de variabilidad los grupos de estudio, el análisis de la varianza y la posterior prueba de F, no se pudo detectar diferencias significativas en los contenidos de Corts en ambos momentos de medición (a. m. [F = 0,14; p = 0,8677]; p. m. [F = 0,04; p = 0,9573]). Al analizar los factores grupo, género y su interacción, ninguna de las fuentes de variación resultó significativa. Al incluir los factores grupo e IMC, se detectaron efectos significativos de interacción para Corts-a. m. (p = 0,0009) y efecto del IMC para Corts-p. m. (p = 0,0215). Se detectó que los individuos con IMC = 1 presentan mayor contenido de Corts-p. m. Al incluir los tres factores (grupo, IMC, género) y sus interacciones en el análisis, no se encontró efecto significativo de ninguno de los factores, solamente en la interacción grupo por IMC para Corts-a. m. (p = 0,0003). 

Con el fin de profundizar en el análisis de los efectos de la cafeina sobre AAs y Corts, se realizó un análisis de componentes principales (ACP) que incluyó como criterio de clasificación los grupos de estudio. No obstante, los ANOVA no han podido detectar diferencias significativas entre los grupos de estudio, el ACP ha logrado reducir la representación de dos dimensiones sin pérdida de variabilidad y, sobre la primera coordenada principal (que conserva el 91,1% de la variabilidad), se observa la diferenciación entre los grupos 1 y 2 respecto del 3. Los dos primeros se encuentran asociados con individuos que presentan mayores contenidos de Corts-p. m., AAs-a. m. y AAs-p. m., mientras que los del Gurpo 3 se relacionan con mayores contenidos de Corts-a. m.

## DISCUSIÓN

En este trabajo de investigación, realizado en adultos jóvenes con actividades diarias normales, el consumo habitual de café no alteró los niveles y patrones diarios de Corts y AAs. En coincidencia con estos resultados, en otros estudios no se observó asociación entre el consumo de cafeína y la actividad de AAs ^11^^,^^17^ y Corts ^12^^,^^18^^,^^19^. Se estipuló que, en ausencia de estrés, el perfil farmacocinético de la cafeína y sus metabolitos no sufren las modificaciones generadoras de alteraciones en la actividad de AAs ^17^^,^^20^. Con respecto al Corts, ha sido demostrado que el consumo regular de cafeína produce efectos de tolerancia y una respuesta de cortisol reducida ^21^.

Sin embargo, la mayoría de los estudios publicados, que han medido los niveles de AAs y Corts en relación con el consumo de café, fueron realizados en presencia de situaciones estresantes. Se ha demostrado que la ingesta diaria de cafeína, junto con la exposición al estrés, se asocia con la activación del SNS y el aumento de los niveles de AAs ^3^^,^^10^^,^^11^. Asimismo, se determinó que la cafeína, en dosis dietéticas, aumenta la secreción de cortisol plasmático y Corts durante una variedad de factores estresantes, debido, en parte, a un aumento en la liberación de hormona adrenocorticotrópica en la hipófisis ^18^^,^^21^^,^^22^. 

Por esa razón, a fin de evitar cualquier factor estresante, en este trabajo se realizó un muestreo cuidadoso de saliva en el hogar con un protocolo riguroso. El automuestreo de saliva se realizó solo en dos tiempos, a pesar de que la medición diurna de cortisol, para ser confiable -según algunos autores-, requiere múltiples puntos de recolección a lo largo del día ^13^. No obstante, la necesidad de obtener muestras de saliva en varias ocasiones puede alterar la rutina, favorecer el estrés y estimular una mayor concentración de Corts ^23^. Asimismo, la relación docente-investigador estudiante, con recopilación de datos bilateral, pudo haber generado confianza en los participantes de esta investigación, tal como se describió en otros contextos ^24^.

No obstante, en condiciones no estresantes, otros autores observaron que el consumo agudo de café activa la AAs ^12^^,^^25^ y el Corts ^22^. Los efectos estimulantes de la cafeína sobre la AAs se asocian con su actividad simpaticomimética ^26^^,^^27^. Con relación al Corts, la cafeína aumenta su secreción mediante la activación de componentes importantes de la respuesta pituitaria-adrenocortical ^21^. Sin embargo, estos efectos agudos se dan en situaciones experimentales con la ingesta de cápsulas que producen niveles máximos de cafeína en segundos, los cuales no se alcanzan cuando se bebe una taza de café durante un período de varios minutos, como sucedió en este trabajo. En consecuencia, es viable que exista un nivel umbral de administración de cafeína necesario antes de que se alteren los niveles de los biomarcadores ^3^. Por ese motivo, en este estudio se controló la ingesta de café antes de las tomas de muestra, a fin de evitar los efectos agudos de la cafeína, que en otros estudios han provocado variabilidad de los resultados ^12^^,^^22^^,^^25^. Así, los estudiantes participaron en su estado de vida habitual, sin privación de café, para evitar los efectos de la abstinencia de cafeína y estrés en la vida cotidiana ^2^.

El consumo de cafeína de cada estudiante depende del número de tazas de café por día. Para la porción de 250 mL de café regular instantáneo, el contenido de cafeína calculado de cada taza es de 75-106 mg de cafeína ^28^^,^^29^. En este trabajo, los estudiantes que excedieron el nivel recomendado de consumo diario de cafeína >400 mg/día ^30^^,^^31^ fueron los consumidores de ≥ 5 tazas de 250 ml de café. Este grupo, en el ACP mostró mayores contenidos de Corts-a. m.. Se ha demostrado que, en adultos sanos consumidores de cafeína a diario, los niveles de cortisol se reducen, pero no desaparecen totalmente, al compararlos con los niveles alcanzados por los consumidores no habituales en dosis repetidas ^22^. 

Con respecto al Corts, las diferencias observadas entre los estudios sobre su respuesta a la ingesta de café, en condiciones no estresantes o en diversos grados de consumo agudo de cafeína, se puede deber a la gran variabilidad intra e interindividual del Corts ^9^, tal como se observó en este estudio, cuyos niveles registraron altos coeficientes de variabilidad. Además, nuestros resultados sobre la falta de efecto del consumo de café sobre el Corts y la AAs pueden deberse a las propiedades antiestrés atribuidas al café ^12^. 

Se conocen diferencias sexuales en la absorción, distribución, metabolismo y tasas de eliminación de la cafeína ^14^ que podrían mediar o moderar los efectos de la cafeína en los niveles de AAs y Corts. Las concentraciones de AAs más altas en los hombres, observadas en este estudio, confirman hallazgos previos ^32^ y se pueden explicar por el predominio simpático relativamente más alto que en las mujeres ^33^.

Este estudio es uno de los primeros, hasta donde sabemos, en investigar los efectos del café sobre el Corts y la AAs en individuos sanos (hombres y mujeres), en condiciones no estresantes y con tomas de muestra salival en el hogar. Sin embargo, se deben tener en cuenta algunas limitaciones al considerar los resultados. Primero, la generalización de los hallazgos del estudio se limita a entornos universitarios similares o a adultos jóvenes sanos, y no representa a toda la población. Otra limitación es el carácter transversal de la investigación, que solo informa asociaciones y no causa-efecto. 

## CONCLUSIONES

El café, consumido en entornos sociales habituales, no afecta la actividad de la AAs y el Corts, al menos en jóvenes que consumen cafeína con regularidad. Los resultados del presente trabajo respaldan resultados de otros estudios, en los cuales se observó que los niveles de cafeína en los consumidores habituales de café no están asociados con la actividad de AAs y Corts. Los hallazgos de este trabajo sugieren que el Corts y la AAs pueden ser utilizados como biomarcadores confiables, para estudios en los que se analizan los efectos de la cafeína sobre la salud.
